# Swimming at Increasing Speeds in Steady and Unsteady Flows of Atlantic Salmon *Salmo salar*: Oxygen Consumption, Locomotory Behaviour and Overall Dynamic Body Acceleration

**DOI:** 10.3390/biology13060393

**Published:** 2024-05-29

**Authors:** Wisdom E. K. Agbeti, Arjan P. Palstra, Suzy Black, Leonardo Magnoni, Martin Lankheet, Hans Komen

**Affiliations:** 1 Animal Breeding and Genomics, Wageningen University & Research, 6700AH Wageningen, The Netherlands; arjan.palstra@wur.nl (A.P.P.); hans.komen@wur.nl (H.K.); 2 Seafood Technologies, The New Zealand Institute for Plant and Food Research Limited, Nelson 7043, New Zealand; suzanne.black@plantandfood.co.nz (S.B.); leonardo.magnoni@plantandfood.co.nz (L.M.); 3 Experimental Zoology Group, Wageningen University & Research, 6700AH Wageningen, The Netherlands; martin.lankheet@wur.nl

**Keywords:** exercise physiology, acoustic sensor tags, offshore aquaculture, wave movements, climate change

## Abstract

**Simple Summary:**

Temperature rises, competition for coastal resources, and stricter regulations are forcing finfish farms to move from sheltered shore locations to more exposed open ocean areas. But knowledge is lacking on how the flow conditions in sea pens in these open areas will affect their physiological performance. To evaluate the swimming performance under steady and unsteady flows at increasing flow speeds, post-smolt Atlantic salmon were induced to swim in a swim tunnel under laboratory conditions. Oxygen consumption and locomotory behaviour were monitored, and overall dynamic body acceleration (ODBA) was recorded by implanted acoustic sensor tags. ODBA correlated strongly with oxygen consumption, allowing the application of such sensor tags to predict energy use. Swimming in unsteady flow is energetically more costly for post-smolt Atlantic salmon than when swimming in steady flow.

**Abstract:**

The swimming performance of cultured finfish species is typically studied under steady flow conditions. However, flow conditions are mostly unsteady, for instance, as experienced in sea pens in exposed sea areas. Using a Loligo swim tunnel, we investigated the effects of swimming in steady and unsteady flows at increasing swimming speeds on post-smolt Atlantic salmon. Oxygen consumption (MO_2_), locomotory behaviour, and overall dynamic body acceleration (ODBA), as determined with implanted acoustic sensor tags, were compared between both flow conditions. Results were obtained for mean swimming speeds of 0.2 to 0.8 m.s^−1^ under both flow conditions. Sensor tags that were implanted in the abdominal cavity had no significant effects on MO_2_ and locomotory parameters. The MO_2_ of fish swimming in unsteady flows was significantly higher (15–53%) than when swimming in steady flows (*p* < 0.05). Significant interaction effects of ODBA with flow conditions and swimming speed were found. ODBA was strongly and positively correlated with swimming speed and MO_2_ in unsteady flow (R^2^ = 0.94 and R^2^ = 0.93, respectively) and in steady flow (R^2^ = 0.91 and R^2^ = 0.82, respectively). ODBA predicts MO_2_ well over the investigated range of swimming speeds in both flow conditions. In an unsteady flow condition, ODBA increased twice as fast with MO_2_ compared with steady flow conditions (*p* < 0.05). From these results, we can conclude that (1) swimming in unsteady flow is energetically more costly for post-smolt Atlantic salmon than swimming in steady flow, as indicated by higher MO_2_, and (2) ODBA can be used to estimate the oxygen consumption of post-smolt Atlantic salmon in unsteady flow in swim tunnels.

## 1. Introduction

Swimming capacity is one of the essential features needed to survive in an aquatic environment. Swimming plays a vital role in searching for food, avoiding predators and unfavourable conditions, and migrating to feeding or spawning grounds. Most fish swim by undulating their bodies and/or fins to create thrust [1,2]. Salmonids use the posterior section of their bodies and their caudal fins to propel themselves by flexing and creating a backward-moving wave, generating thrust for swimming [1,2,3,4]. Aside from basal metabolic rate and specific dynamic action, swimming exercise is believed to constitute a significant portion of a fish’s energy expenditure [5,6,7]. The energy expenditure for moving a specific distance at a particular velocity is not fixed and can fluctuate depending on the environmental circumstances and the individual’s, as well as the species’, characteristics [8,9,10].

The estimation of energy expenditure during swimming, also known as active metabolic rate, involves measuring oxygen consumption (MO_2_) under controlled conditions, commonly in a swim tunnel [11,12,13,14]. This estimation frequently demonstrates a strong positive correlation with swimming speed and locomotory parameters such as tail beat frequency (TBF), which has been adopted as an indicator of swimming speed in fish [15,16]. However, most available oxygen consumption data and the relationship with swimming speed and locomotory parameters, have been obtained from studies on fish swimming in steady flows [16,17,18]. Water flow patterns in nature are typically complex, encompassing both turbulence (vortices of varied strengths and sizes) and unsteady flow (variable alternations of flow velocity and direction) [19]. Under natural conditions, or semi-natural conditions as experienced in aquaculture sea pens, fish mostly experience unsteady flows. Unsteadiness of water flow will influence the energetic costs of locomotion [20,21] and can affect the schooling assemblies and swimming speeds of cultured fish species [22] to varying degrees. A study carried out by Hoop et al. [23] showed that unsteady water flows can significantly affect the swimming performance and energetics of shiner perch *Cymatogaster aggregate* during station holding. Low-amplitude, unsteady flow resulted in reduced swimming efficiency and increased energy expenditure. Roche et al. [24] also found that shiner perch swimming in highly unsteady flow have consistently higher oxygen consumption rates (by 25% on average) than those swimming in steady flow.

In the light of climate change and environmental sustainability challenges, marine fish farms are compelled to move from traditional near-shore sites to more exposed open ocean areas [25]. These offshore locations are characterised by unsteady flow conditions, which deviate from the relatively stable environments that near-shore fish farms typically offer. However, it is unknown how the flow conditions in these open ocean areas will affect the swimming capabilities, behaviour, and overall energy expenditure of farmed finfish like Atlantic salmon (*Salmo salar* Linneaus 1758). In uncontrolled open aquatic environments, fish and other aquatic organisms display wide variations in energy usage, along with notable inter-species behavioural differences [26]. However, linking energy usage to physiological and behavioural characteristics remained challenging, mainly due to difficulties in monitoring energy expenditure in such environments. Therefore, understanding the swimming behaviour of free-swimming fish in unsteady flows and its correlation with energy expenditure is crucial. This understanding is not only essential for higher-order ecological processes such as ecosystem functioning but also vital for comprehending organism mobility, interactions, and disturbance response patterns as experienced in both natural and controlled environments [22,26,27].

With advancements in biotelemetry, implantable sensor tags are emerging as promising and innovative tools to monitor species-specific acceleration patterns in both natural environments and aquaculture settings, providing insights into an animal’s mechanical energy expenditure [28,29,30,31,32,33]. Energy expenditure resulting from movement is regulated by muscle contractions that generate accelerations in the body [28,32]. Therefore, monitoring the tri-axial acceleration of fish can serve as a valuable indicator for activity-related energy expenditure. Acoustic transmitters equipped with tri-axial accelerometer sensors, for instance, have been surgically implanted or externally attached to fish to assess the overall dynamic body acceleration (ODBA) and used as a proxy for energy metabolism [31,32]. ODBA is based on the idea that when the effects of gravity are removed, the remaining acceleration in the animal’s movement is solely related to its own activity and is directly proportional to the amount of adenosine triphosphate used during muscle contractions [32,34]. Very recently, Zrini and Gamperl [35] reported that acceleration substantially increased with the swimming speed of adult Atlantic salmon swimming in a swim tunnel under steady flow conditions, but these authors did not investigate the relation to MO_2_. Wilson and his colleagues [31] found a positive correlation between MO_2_ and ODBA in wild adult sockeye salmon under steady flow conditions. But it is unknown how this relationship will be affected by unsteady flow conditions.

In this study, we focus on unidirectional flows that are unsteady in speed, reflecting the wave movements that fish will experience in offshore aquaculture system designs. Using post-smolt farmed Atlantic salmon, one of the main marine farmed fish species, we investigated the relationship between oxygen consumption, locomotory behaviour, and ODBA when swimming at increasing speeds in a swim-tunnel, comparing unsteady with steady flow conditions. We hypothesise that, for salmon, acceleration tags can be used as a proxy for activity-related energy expenditure. Because unsteady flow will require a constantly alternating pattern of acceleration and deceleration, we hypothesise that swimming in unsteady flow will increase acceleration and therefore be energetically more costly.

## 2. Materials and Methods

### 2.1. Ethics

Experimental protocols complied with the current laws of The Netherlands and were approved by the Central Committee for Animal Experiments (CCD), project number AVD401002016652, 12 December 2016, and by the Animal Experiments Committee (DEC) and Authority for Animal Welfare (IvD), experiment number 2016.D-0039.005, 1 September 2021, of Wageningen University.

### 2.2. Experimental Fish

Atlantic salmon smolts in the range of 130 to 400 g were provided by Aquafuture (Hagen, Germany) and transported to the Wageningen University and Research Animal Experimental Facilities (CARUS, Wageningen, The Netherlands). Upon arrival, fish were allowed to acclimatise for 14 days in three circular holding tanks with a volume of 1000 L each with a shelter area, supplied with well-aerated brackish water at 14 ± 1 °C, and connected in a recirculating aquaculture system. Within 2 weeks, salinity was gradually increased from 15 to 34 ppt. During acclimation, fish were kept in a 12 h light–dark regime and hand-fed with a commercial pellet (crude protein 43%, ether extract 29%, ash 7%, 3 mm). After the acclimation period, feeding was done using automatic belt feeders, and the water temperature was gradually reduced from 14 ± 1 °C to 12 ± 1 °C with 0.5 °C per day.

### 2.3. Swim Tunnel, Flow Measurements, and Calibration

A Loligo swim tunnel (#SW10150, Loligo Systems, Viborg, Denmark) with a total volume of 30 L and a swim section of 9 L (46 cm length × 14 cm width × 14 cm height) was used to conduct this experiment. The swim tunnel was submerged in a water bath to maintain a water temperature of 12 °C. An AC motor (DRS71S4/FI/ACE1/EI72, SEW-EURODRIVE, Greve, Denmark) drove a propellor to generate the required flows. Within the swim tunnel, a flow straightener (0.6 cm in diameter) ensured laminar flow, and a double grid (0.3 × 0.3 cm and 1.2 × 1.2 cm) at the end of the swim section prevented fish from escaping the swim section. Flow speed within the swim tunnel was regulated externally via a speed control box (0.5 HP MOVITRAC LTE, SEW-EURODRIVE, Devilliers Way, Trident Park, Normanton, West Yorkshire, WF6 1GX, UK) using a Python-based computer program. The computer program controlled a voltage signal generated by a national instruments board (NB usb 6211), driving the speed controller. Mean flow speeds were calibrated by measuring speeds with a downward-faced Vector Acoustic Doppler Velocimeter (ADV; Nortek AS, Rud, Norway) in the working section of the swim tunnel. A calibration graph was generated for voltages ranging from 0.4 to 4 V (covering the full dynamic range of the controller), in steps of 0.1 V (Figure S1).

In our study, we focused on flows that are unsteady in speed, reflecting the wave movements that fish will experience in offshore settings. To mimic this unsteady flow, we applied sinusoidal modulations of flow speed around a constant mean flow speed. Measurements were performed at a fixed frequency of 1/12 Hz, corresponding to a wave period of 12 s, which is similar to the time to complete one offshore wave cycle. The amplitude of modulations was set to 0.1 m.s^−1^, which was calibrated by measuring flow speed modulations at a range of amplitudes for the control signal at each mean flow speed level (0.2–1 m.s^−1^). Flow speeds were recorded at high temporal resolution, and a sinusoid was fitted to the data (Figure S1).

### 2.4. Respirometry

To determine the MO_2_, in the form of active metabolic rate, oxygen concentrations in the swim tunnel were measured using an oxygen probe (DAQ-PAC-G4; Loligo Systems Aps, Viborg, Denmark). From the decline of O_2_ concentration as a percentage of the total O_2_ amount (∆O_2_%), the O_2_ consumption rate (MO_2_; in mg O_2_ kg^−1^ h^−1^) and cost of transport (COT; in mg kg^−1^ km^−1^) were calculated with the following equations:

(1) MO2=(∆O2(DOmax∗V100)BW∗t

where $∆O_{2}$ is percentage oxygen saturation; DOmax (mg L^−1^) is the maximum amount of oxygen dissolved in the seawater; V is the volume of the swim tunnel (30 L); BW (kg) is the body weight of the fish; and t is the time in hours.

(2) $$\mathrm{COT}=\frac{MO_{2}}{U}$$

where MO_2_ is the consumed oxygen (mg O_2_ kg^−1^ h^−1^), and U is the speed in kilometres per hour (km h^−1^).

### 2.5. Swimming Exercise Protocol

The swim tunnel was filled with system seawater at 34 ppt and 12 ± 1 °C and kept recirculating using an Eheim pump with a capacity of 40 L.min^−1^. Experimental fish, starved for 24 h prior to the trial, were randomly selected from the holding tank and anesthetised in aerated system water containing 0.3 mL.L^−1^ phenoxyethanol. When anesthetised, the fish was weighed to determine body weight (BW), total and standard length (TL and SL, respectively), and Fulton’s condition factor (K; [36]) was calculated. Individual fish were then transferred into the swimming section of the respirometer, and the lid was tightened. The inlet and outlet valves of the swim tunnel were simultaneously opened, so water was flowing through during fish recovery and acclimation for one hour. A critical swimming speed (U_crit_) protocol was then executed starting at 0 m.s^−1^, still without propeller activity, and then from 0.2 up to 1.0 m.s^−1^, with increments of 0.2 m.s^−1^, and swimming for 30 min at each speed. During the whole test, the oxygen content of the swim tunnel was measured at a frequency of 1 measurement per second. The swim trial was terminated when a fish fatigued, determined as the point when the fish touched the rear metal grid of the swim section for more than 20 s and could not be stimulated to swim within this period. The fish was then removed from the test chamber and transferred to a recovery tank. The exact time of fatigue was recorded and used to calculate the critical swimming speed (U_crit_), according to Brett [11] and Plaut [37].

U_crit_ = U_i_ + [U_ii_ (T_i_/T_ii_)] (3)

where U_crit_ is the critical swimming speed in m.s^−1^, (absolute U_crit_), U_i_ is the highest velocity completed before exhaustion in m.s^−1^, U_ii_ is the prescribed velocity increment in m.s^−1^, T_i_ is time to fatigue at the final velocity level in minutes, and T_ii_ is the prescribed time interval (=30 min).

Optimum swimming speed (U_opt_) was determined by plotting a two-degree polynomial trend line through COT values vs. swimming speeds. The point on this trend line with the lowest COT was calculated by equalising the first derivative to zero [38]. MO_2_ was calculated from the oxygen measurements for the 15 min periods during each swimming speed. Background oxygen consumption was determined following the same U_crit_ protocol, but without fish, and values were used to subtract from the oxygen consumption of the fish. The solid blocking effects of the fish were calculated following Bell and Terhune [39].

### 2.6. Locomotory Behaviour

High-speed video footage of salmon locomotion in the swim tunnel was recorded using a Basler 2040-90um NIR USB3 camera mounted one metre above the centre of the swim section. The camera’s field of view was adjusted to cover the entire swim section, and video was recorded at a frame rate of 25 frames per second with a 15 ms exposure time. To improve the sensitivity by a factor of 4, pixels were binned at 2 × 2. The final images had a resolution of 14.25 pixels per cm and were 1024 × 512 pixels in size in total. Real-time fish contour detection was performed using custom software developed in Python, which utilised the OpenCV image analysis library (see Figure 2 in [16]). Fish detection was achieved through a series of image processing steps, including a median (3 pix) and Gaussian blur (5 pix) filter to reduce noise, histogram normalisation to enhance image contrast, and a luminance threshold to distinguish dark fish from a light background.

After detecting objects using the “find contours” routine, the fish was selected based on surface area and the length-width ratio of an ellipse fitted to the contour. A standard Kalman filter in OpenCV was utilised to obtain smoothed estimates of fish tracks, which were quantified by the centre of mass of the contour, along with timing information and Kalman x-y locations. Full body contours were saved for later analysis. To analyse the midline of each fish, a distance transform was used to determine the nearest distance to the contour for each pixel. The head location and width were obtained by fitting a line to all points with a distance greater than 80% of half the body width, producing a strip of “midline” points in the fish’s anterior region (see Figure 2 in [16]). The snout was identified by detecting the first point outside the contour on a line fitted to the midline points.

To create the complete axis of the fish, the ridge of the distance transform’s maxima was traced, starting from the snout in increments of 0.7 cm. To track the maxima, we repeatedly identified the maximum point on a circle with a 0.7 cm radius around the previous point and then cleared the values within the circle to prevent directional reversals. Tracking was halted when the tail tip was reached. The resulting axis was then slightly smoothed using a univariate spline, separately for x and y data, with a spline order of 3 and a smoothing factor of 5, to reduce the impact of any contour irregularities on the distance transforms.

We determined tailbeat parameters by selecting a point in the tail that was 14.0 cm away from the snout and measuring its lateral excursion relative to the midline through the head. We obtained tailbeat frequency (TBF) and amplitude (TBA) by analysing the tail excursion as a function of time through spectral analysis.

Spectrograms were created by calculating temporal windows with a size of 1.28 s (32 frames), which were then shifted frame by frame. To increase frequency domain resolution, the signal was padded with zero values to a width eight times that of the original signal. Frequency and amplitude were determined at each frame by identifying the maximum value in the spectrogram. A similar approach was used to calculate head width frequency (HWF) and amplitude (HWA) as a proxy for opercular movements. HWF represents the complete oscillation of the head region within a given time period. HWA measures how much the head region moves from its maximum position in one direction to its maximum position in the opposite direction during a single cycle.

### 2.7. Overall Dynamic Body Acceleration

Eleven days prior to the first swim experiment, acoustic accelerometer transmitters with ODBA settings (Thelma Biotel Ltd., Trondheim, Norway; model A-LP7; diameter size: 7.3 mm; length: 17 mm; weight in air: 1.9 g; weight in water: 1 g; power output: 139 dB; sampling frequency: 25 Hz; transmission frequency: 71 kHz; transmission interval: 30–50 s) were implanted into N = 20 individuals. Fish were randomly hand-netted from the holding tank and anesthetised as described before. When anesthetised, each fish was weighed and length was measured. The fish was then placed on a surgical table with the ventral side up for implantation of the sensor tag. This was done under a continuous flow of water with a low dose of phenoxyethanol (0.15 mL.L^−1^) passing over the gills. Before each incision, the surgical equipment was rinsed in 70% ethanol and allowed to dry. An incision (∼1 cm) was made on the ventral surface, posterior to the pelvic girdle. The transmitter was gently passed through the incision into the body cavity, just dorsally from the pelvic girdle. For identification, a passive integrated transponder (PIT-tag; Biomark^®^ Inc, Boise, ID, USA) was also implanted through the incision before closing it with a silk suture. After the surgical procedure (handling time: 5–7 min), the tagged fish was transferred to a quarantine tank for recovery. Prior to the start of the swim test, a watertight opening was made in the closing lid of the working section to hold firmly the snout of the acoustic receiver (Thelma Biotel Ltd.,Trondheim, Norway; model TBR 700; diameter: 75 mm; length: 230 mm). This was positioned close to the straightener to record the transmitter’s signals during the experiment. The acoustic transmitter with ODBA settings uses the sum of three-dimensional body acceleration (Ax = surge, Ay = sway, Az = heave) and extracts only dynamic acceleration (due to movement) by subtracting static acceleration (due to gravity) from the raw acceleration data using an inbuilt algorithm before transmitting the data acoustically to a receiver (Thelmabiotel, Trondheim, Norway; https://www.thelmabiotel.com/outputs/activity/ accessed on 6 March 2023). ODBA was then calculated by summing the dynamic acceleration values for each axis.

### 2.8. Experimental Groups

Four groups of salmon were used in this experiment: groups 1 and 2 (each N = 10 fish) were non-tagged fish used to measure the MO_2_ and assess locomotory behaviour under unsteady and steady flow conditions, respectively, as described in Section 2.3. Groups 3 and 4 (each N = 10 fish) were the tagged fish that were implanted with acoustic acceleration sensor tags (described in Section 2.7) to quantify ODBA in relation to swimming speed, MO_2_, and locomotory behaviour, under unsteady and steady flow conditions, respectively, as described in Section 2.3.

### 2.9. Statistics

Data were analysed using the R statistical package software, version 4.3.0. R packages lme4 [40] and lmerTest [41] were employed to fit models. All data were tested for normality and homoscedasticity. A one-way analysis of variance (ANOVA) with a Tukey comparison test was performed to assess potential differences in BW, SL, and K between groups. In cases where model assumptions were not met, data were either log-transformed or a non-parametric, the Kruskal–Wallis test with Dunn multiple comparison (Bonferroni method) was used to assess differences.

A linear mixed model (LMM), fitted using restricted maximum likelihood (REML), was employed to examine differences in respirometry and locomotory parameters among fish subjected to unsteady and steady flow conditions. The general model was:

y = X*β* + Zb + *e*.

Here, y represents the vector for one response variable, comprising MO_2_, COT, ODBA, TBF, TBA, HWF, and HWA; X is the design matrix for the fixed effects coefficients *β*, encompassing swimming speed, flow conditions, and tag; Z is the design matrix of the random effects coefficient b, representing individual fish; and *e* is the vector of random errors. We assumed that the random effect and random errors were mutually independent, normally distributed, and identical. The interaction of fixed effects was tested and included in the model when found to be significant. LMM was also used to test whether ODBA was a good predictor of MO_2_. Individual variability was accounted for a random effect in all models. The results were considered significant at *p* < 0.05. All values are reported as means ± SE.

## 3. Results

### 3.1. Biometrical Parameters

The biometrical parameters (BW, SL, and K), as measured at the start of each swim test, for the fish of each experimental group (N = 10), are shown in Table 1. A significant difference was found in BW between tagged and non-tagged fish, both for those that swam in steady flow (*p* < 0.05) and in unsteady flow (*p* < 0.05). SL values were only significantly different for those that swam in unsteady flow (*p* < 0.05). At the moment of tagging, the fish weighed 268 ± 6 g and measured 27 ± 1 cm. These data show that tagged fish did not grow significantly from the moment of implantation until the moment of the swim test, in contrast to the fish that were not tagged. Because of the resulting difference in size between tagged and non-tagged fish, BW and SL were included in the LMM as covariates.

### 3.2. Respirometry

#### 3.2.1. Critical Swimming Speed

No consistent differences between groups could be detected in U_crit_. Under steady flow conditions, five tagged fish fatigued at 0.8 m.s^−1^ with a mean U_crit_ of 0.78 ± 0.01 m.s^−1^, while the remaining five fatigued at 0.6 m.s^−1^ with a mean U_crit_ of 0.61 ± 0.01 m.s^−1^. In the non-tagged group under similar flow conditions, six fish fatigued while swimming at 0.8 m.s^−1^ (U_crit_ = 0.71 ± 0.03 m.s^−1^) and four fish fatigued at 1.0 m.s^−1^ (U_crit_ of 0.96 ± 0.03 m.s^−1^). During unsteady flow conditions, only one tagged fish achieved a U_crit_ of 0.95 m.s^−1^, while the other nine tagged fish fatigued at a mean U_crit_ of 0.79 ± 0.04 m.s^−1^. In the non-tagged group under these conditions, seven fish displayed a mean U_crit_ of 0.62 ± 0.01 m.s^−1^, and three fish exhibited a mean U_crit_ of 0.77 ± 0.01 m.s^−1^. Solid blocking effects were found to be negligible, so flow speeds were not adjusted.

#### 3.2.2. Oxygen Consumption

**Figure 1:**
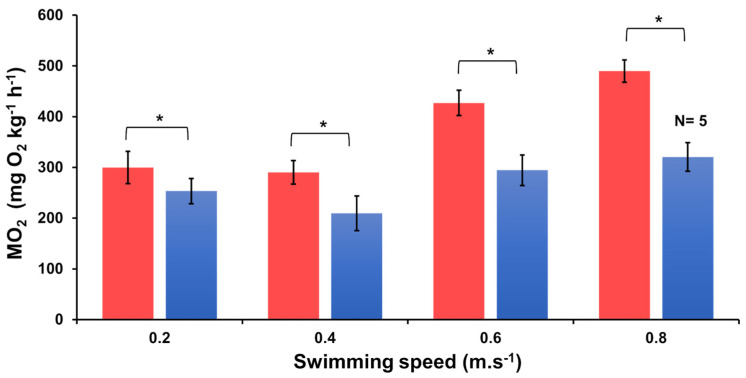
Oxygen consumption (MO_2_) of tagged fish (*Salmo salar*) at increasing swimming speed in both steady and unsteady flow. The blue colour represents swimming in steady flow, and the red colour represents swimming in unsteady flow. Each bar represents N = 10 fish, unless indicated otherwise, because fish had fatigued. A similar graph for non-tagged fish is included in Supplementary Figure S2. An asterisk indicates a significant difference (LMM *p* < 0.05) between steady and unsteady flow. MO_2_ values are shown as means ± SE.

In general, oxygen consumption remained similar at the lower swimming speeds of 0.2 and 0.4 m.s^−1^ (Figure 1). At 0.6 m.s^−1^, MO_2_ for fish in both experimental groups was significantly higher than at the lower speeds (Table S1). These values then remained similar for fish swimming at 0.8 m.s^−1^, probably because fish that could not maintain swimming fatigued. No differences were found in oxygen consumption between the tagged and non-tagged fish that swam in either unsteady or steady flow (LMM, *p* = 0.2; Table S1).

Significantly higher MO_2_ values were observed in tagged fish swimming in unsteady flow as compared with tagged fish that swam in steady flow (Figure 1), as well as between non-tagged fish (Figure S2) that swam in unsteady vs. steady flow (LMM, *p* < 0.05). The impact of swimming speed at 0.6 m.s^−1^ exhibited a statistically significant and positive effect (*p* < 0.05), as well as at 0.8 m.s^−1^ (*p* < 0.05) compared with 0.2 m.s^−1^. Tag implantation had no effect on MO_2_ (Table S1). Flow condition, swimming speed, and tag implantation (fixed effects) accounted for a substantial amount of variation observed in the MO_2_ of the fish used in this study (R^2^ marginal = 0.46; Table S1).

The MO_2_ values of tagged fish swimming in unsteady flow (Figure 1) ranged from 300 ± 32 mg O_2_ kg^−1^ h^−1^ when swimming at 0.2 ± 0.1 m.s^−1^ (N = 10) to 490 ± 22 mg O_2_ kg^−1^ h^−1^ when swimming at 0.8 ± 0.1 m.s^−1^ (N = 10). These values were 15% to 53% higher than the MO_2_ values recorded by tagged fish that swam in steady flow, ranging from 253 ± 25 mg O_2_ kg^−1^ h^−1^ when swimming at 0.2 m.s^−1^ (N = 10) to 320 ± 28 mg O_2_ kg^−1^ h^−1^ at 0.8 m.s^−1^ (N = 5; note that the latter value may be an underestimation due to fatigue of half of the fish).

Similarly, MO_2_ values of non-tagged fish (Figure S2) were 314 ± 18 mg O_2_ kg^−1^ h^−1^ when swimming at 0.2 m.s^−1^ (N = 10), and up to 501 ± 39 mg O_2_ kg^−1^ h^−1^ when swimming at 0.8 m.s^−1^ (N = 3) in unsteady flow. In steady flow, MO_2_ values ranged from 241 ± 27 mg O_2_ kg^−1^ h^−1^ when swimming at 0.2 m.s^−1^ (N = 10), up to 407 ± 39 mg O_2_ kg^−1^ h^−1^ at 0.8 m.s^−1^ (N = 10, with all fish except for one fatiguing after swimming 11 min at this speed).

#### 3.2.3. Cost of Transport and Optimal Swimming Speed

COT decreased significantly with increasing swimming speed in both unsteady and steady flows (LMM, *p* < 0.05) until reaching the optimal swimming speed (Table S1). COT differed significantly between swimming in unsteady and steady flow at each flow speed (LMM, *p* < 0.05).

COT of tagged fish in unsteady flow ranged from 416 ± 44 mg O_2_ kg^−1^ km^−1^ when swimming at 0.2 m.s^−1^ to an extrapolated COT_min_ of 158 mg O_2_ kg^−1^ km^−1^ when swimming at U_opt_, while in steady flow the values were 352 ± 34 to 100 mg O_2_ kg^−1^ km^−1^. Similarly, COT of non-tagged fish, swimming in an unsteady and steady flows at 0.2 m.s^−1^ to U_opt_ ranged from 436 ± 25 to 167 mg O_2_ kg^−1^ km^−1^, and from 335 ± 38 to 136 mg O_2_ kg^−1^ h^−1^, respectively. A similar U_opt_ was found for fish swimming in either unsteady or steady flow. Comparatively, U_opt_ for fish of each of the four experimental groups were in the same range (0.65–0.68 m.s^−1^) without any significant differences. Higher COT_min_ values were found for swimming in unsteady flow.

### 3.3. Swimming Behaviour

#### 3.3.1. Tail Beat Frequency

**Figure 2:**
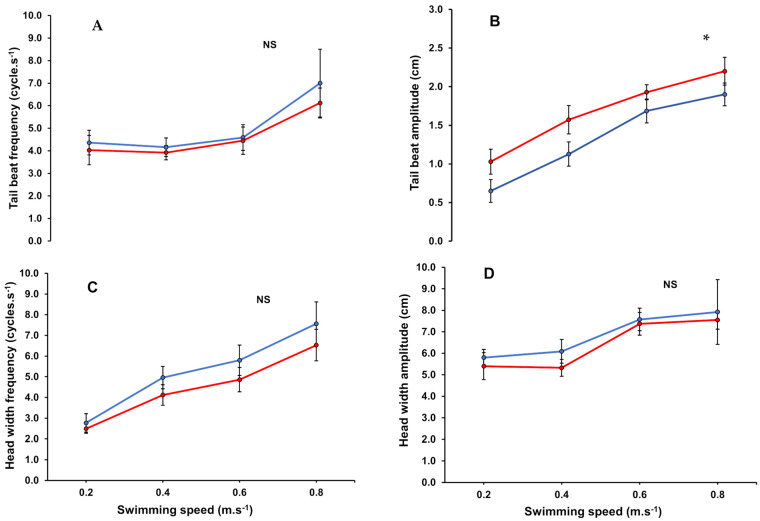
Tail beat frequency, tailbeat amplitude, head width frequency, and head width amplitude vs. swimming speed for tagged post-smolt Atlantic salmon (*Salmo salar*) swimming in steady and unsteady flow with (**A**) tail beat frequency vs. swimming speed; (**B**) tail beat amplitude vs. swimming speed; (**C**) Head width frequency vs. swimming speed; and (**D**) head width amplitude vs. swimming speed. Non-tagged fish showed similar relationships (Figure S4). The solid blue line represents swimming in steady flow, while the solid red line represents swimming in unsteady flow. Asterisk indicates a significant difference (LMM *p* < 0.05) between steady and unsteady flow, while NS indicates no significant difference.

Tail beat frequency (TBF) of tagged fish increased from 4.36 to 7.02 cycles.s^−1^ when swimming in steady flow and from 4.03 to 6.12 cycles.s^−1^ in unsteady flow, with swimming speeds ranging from 0.2 to 0.8 m.s^−1^ (Figure 2A). Non-tagged fish also showed similar response curves under both flow conditions (Figure S3). From 0.4 to 0.8 m.s^−1^, the relationship between TBF and swimming speed was described by a linear function (Table S2). Only at a higher swimming speed of 0.8 m.s^−1^, we observed a significant increase in TBF values of fish in both steady and unsteady flow (Table S1). However, no significant differences were observed between flow conditions (Figure 2A), although unsteady flow tends to have a negative effect on TBF (Table S1). The various ranges of TBF values for each experimental group obtained from the high-speed camera are shown in Table S2.

#### 3.3.2. Tail Beat Amplitude

Tail beat amplitude (TBA) increased significantly with increasing swimming speed (Figure 2B, Figure S3, Table S1). TBA values of tagged fish (Figure 2B) swimming in unsteady flow were significantly higher than tagged fish swimming in steady flow when compared at each of the swim speeds (*p* < 0.05). The relationship between TBA and swimming speed among all experimental groups was best described using a power function, showing a correlation coefficient above 0.92 (Table S2). More so, the explanatory power of the fixed effects (swimming speed and flow type) on TBA was also high (R^2^ marginal = 0.55; Table S1).

#### 3.3.3. Head Width Frequency

We detected a significant linear increase in head width frequency (HWF) with increasing swimming speed (Table S1) for fish swimming either in unsteady or steady flow (Figure 2C). Unsteady flow conditions had no significant influence on HWF (LMM, *p* > 0.05). At higher swimming speeds, we observed that fish swimming in unsteady flow showed lower HWF values as compared with fish swimming in steady flow (Figure 2C; Table S1).

#### 3.3.4. Head Width Amplitude

The head width amplitude (HWA) of fish swimming in either unsteady or steady flow increased linearly with swimming speed (Figure 2D). However, no statistical and consistent differences in HWA were found between the unsteady and steady flow conditions (Table S1). Higher swimming speeds of 0.6 and 0.8 m.s^−1^, significantly increased HWA irrespective of flow conditions (Table S1).

### 3.4. Overall Dynamic Body Acceleration

**Figure 3:**
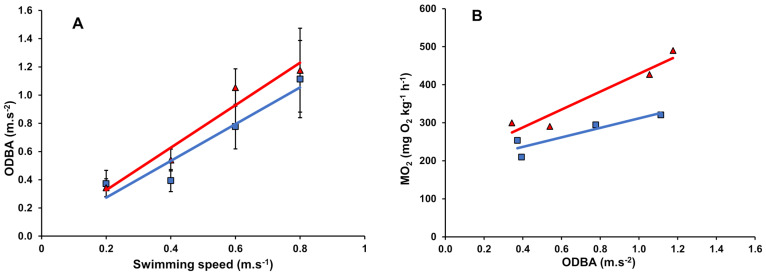
Linear regression between (**A**) overall dynamic body acceleration (ODBA) vs. swimming speed and (**B**) oxygen consumption (MO_2_) vs. ODBA of tagged post-smolt Atlantic salmon (*Salmo salar*). The solid blue colour represents swimming in steady flow, and the solid red colour represents swimming in unsteady flow. Each point represents N = 9 fish for steady flow and N = 9 fish for unsteady flow. Linear functions of ODBA vs. swimming speed under steady and unsteady flow are described by y = 1.30x + 0.01 and y = 1.51x + 0.03, respectively.

A positive linear correlation was observed between ODBA and swimming speed for fish swimming in both unsteady (linear fit: R^2^ = 0.94) and steady (linear fit: R^2^ = 0.91) flows (Figure 3A). When we used a LMM to predict ODBA based on swimming speeds and flow conditions, incorporating an interaction effect between swimming speed and flow condition, we found significant interaction effects of swimming speed and flow condition on ODBA (Table S1).

Further, we used the interaction between swimming speed and flow condition as a covariate in LMM to predict MO_2_ using ODBA as the main predictor variable, still accounting for individual variability (Table S1). We found ODBA to be a significant predictor of MO_2_ (*p* < 0.05; Table S1). ODBA was positively correlated with MO_2_ under both steady (linear fit: R^2^ = 0.82, MO_2_ = [(125 ± 41) × ODBA) + (186 ± 30)]; n = 24, df = 22) and unsteady (linear fit: R^2^ = 0.93, MO_2_ = [(236 ± 47) × ODBA + (193 ± 40)]; n = 24 df = 22) flow conditions (Figure 3B), with a significant regression slope in unsteady flow almost twice (1.9 times) as high as in steady flow (Student *t*-Test, *p* = 0.01, df = 44, t = 2.87).

## 4. Discussion

In nature, fish mostly experience unsteady flows, unpredictable alternations of flow velocity and direction [19]. These environmental factors, in addition to temperature, can impact the metabolic rates and behaviour of fish, leading to alterations in behaviour [26,27] and swimming patterns [22]. But the extent to which it may affect the swimming performance of post-smolt Atlantic salmon is hardly known. This study quantified oxygen consumption, locomotory behaviour, and ODBA and linked ODBA to oxygen consumption for post-smolt Atlantic salmon swimming in steady and unsteady flow. The trials were executed with fish that were implanted with acoustic acceleration tags, as compared with control fish without tags, swimming in a Loligo swim tunnel at increasing swimming speeds.

A first concern in using sensor tags for fish monitoring is the potential impact that these attached or implanted tags may have on the swimming performance and behaviour of fish, which is largely unknown [42]. The sensor tags that were implanted 11 days before the initial swim test in this present study had no effect on oxygen consumption, swimming performance, or the behaviour of Atlantic salmon since no differences were found in the performance and behavioural parameters evaluated between the tagged and non-tagged fish. Other studies in salmonid species that used implanted tags support our results that no alterations occur in swimming performance and behaviour [18,43,44]. Nevertheless, recovery from the surgical procedure led to a disparity in body weight observed between tagged and non-tagged fish. This is in contrast to the findings of Moore et al. [45], who reported that implanting tags in juvenile Atlantic salmon had no impact on their growth during the initial 28 days. Moreover, they noticed that fish resumed normal feeding behaviour within 8 h after the tagging process. The same authors also observed that Atlantic salmon parr and smolts showed no negative impacts of the sensor tags on swimming performance within the first 2 weeks after tagging. On the other hand, Adams et al. [46] reported a temporary reduction in swimming performance for juvenile Chinook salmon 1 day after tagging, but this effect had disappeared after 21 days, which they attributed to the short-term impact of postoperative stress. Føre and his colleagues [47] showed that adult Atlantic salmon (55.5 ± 5.7 cm fork length, mean weight 2100 g) required 4 to 6 days of recovery before useful data collection. In our study, the time from tagging to the swim test ranged from 11 to 21 days for individual fish. Because of this variation in recovery time, we did not anticipate any significant growth differences between the tagged and non-tagged fish. Nevertheless, it is important to note that the implanted tags used in our study adhered to the maximum 2% tag-to-body mass guideline commonly employed to assess the impact of electronic tags on fish in numerous telemetry studies [42,48].

Generally, salmonids in marine waters encounter unsteady flow speeds created by waves. Yet, the extent to which this would affect their swimming capabilities, behaviour, and ultimately energy expenditure is not well known. Most swimming exercise studies on salmonids were carried out under steady flow conditions where oxygen consumption increased exponentially with increasing swimming speed [14,17,49,50]. In our study, we mimicked unidirectional, unsteady flow that varied in speed, reflecting the wave movements that fish will experience in off-shore aquaculture system designs (Figure S1). A sinusoidal wave pattern with a wave amplitude of 0.1 m.s^−1^ and a wave period of 12 s, mimicking the wave conditions in New Zealand offshore areas, was used in this study. This was done in a protocol with swimming speeds ranging from 0 up to 1 m.s^−1^, with increments of 0.2 m.s^−1^, and durations of swimming of 30 min per speed. Swimming performance was compared with steady swimming using the same protocol. We observed that the MO_2_ of individual fish remained fairly similar at lower swimming speeds but increased at higher swimming speeds (0.6 and 0.8 m.s^−1^) in both unsteady and steady conditions (see Figure 1). Such higher oxygen demand at higher swimming speeds is known to increase stroke volume and, subsequently, cardiac output and maximum power output in salmonids [51]. Coping with this high energy demand at higher speeds was also evident in the locomotory behavioural parameters that showed significant increases, especially at the higher swimming speeds, regardless of whether flow conditions were steady or unsteady (Figure 2, Table S1). When swimming in steady flow, our recorded MO_2_ values were comparable to those reported by Hvas and colleagues [17] for Atlantic salmon weighing 437 ± 16 g. In their study, flow speed was gradually increased with steps of 0.15 m.s^−1^ every 30 min until fish reached fatigue. When swimming in unsteady flow, we found that MO_2_ was significantly higher (15% to 53%) than in steady flow. Furthermore, the higher energy use of fish swimming in unsteady flow becomes evident from the higher COT_min_ values in contrast to those swimming in steady flow (Table S1). The difference in energy expenditure between swimming in steady and unsteady flow can be caused by the accelerations and decelerations in conformity to the sinusoidal wave that was created. Body and caudal fin swimmers, such as post-smolt Atlantic salmon, typically exhibit steady swimming interspersed with intermittent bursts and coast gaits [1,4]. Steady swimming is mainly supported by the skeletal slow, or red, muscles, fuelled aerobically, while bursts are enabled by the fast, or white, muscles [2,3]. It is plausible that the accelerations and decelerations and absence of steady swimming behaviour caused by the constant wave-like unsteady flow conditions decrease the swimming efficiency and consequently increase oxygen demand [52]. When swimming speeds increased, we also noted a substantial increase in TBA under unsteady flow conditions as compared with steady flow (Figure 2B). These observations suggest that fish navigating in unsteady flow may need to increase their TBA as the flow velocity rises to counteract the growing drag force, which increases proportionally with the square of the flow speed [1,4]. This additional effort can contribute to the observed increase in energy consumption among fish swimming in unsteady flow [21,24,53]. Thus, wave-like unsteady flow will increase the energy costs of swimming, which should be accounted for when exposing fish in sea pens located offshore.

To better understand the energy expenditure of swimming in unsteady flow, fish were implanted with accelerometer-equipped sensor tags. These tags are purpose-built to capture fish movement patterns in three dimensions, providing valuable information on swimming activity. Nonetheless, it is crucial to conduct laboratory-derived calibrations to understand the attributes and configurations of accelerometer sensor tags tailored to various species and specific environmental circumstances, such as unsteady flow conditions. In our study, we tried to gain insight into the extent to which ODBA may reflect the MO_2_ of Atlantic salmon swimming in unsteady flow, which would enable the use of acceleration sensor tags for estimating energy expenditure under such conditions. To provide greater behavioural resolution to aid in the accurate estimation of MO_2_, we used the ODBA-based accelerometer sensor tag at a frequency of 25 Hz in our study. While lower sampling frequencies in previous studies [31,54] estimated activity level and swimming speed, it may introduce bias in metabolic rate estimation due to the high variations in output below 5 Hz [55]. Our results showed an increase in ODBA values with increasing swimming speed (Figure 3A), as observed with MO_2_ (Figure 1), and all four locomotory parameters evaluated under these two flow conditions (Figure 2). As such, ODBA predicts MO_2_ well over a wide range of swimming speeds (see Figure 3 and Table S1). The strong and positive linear correlations between ODBA and MO_2_ in steady and unsteady flow (R^2^ values > 0.82; Figure 3B) observed in this study provide support for the concept that ODBA can serve as a reliable proxy for quantifying the energy expended during locomotion. The slope estimates of the linear regression between ODBA and MO_2_ under both flow conditions in our study were statistically different (twice as high in unsteady flow than in steady flow) (Figure 3B). This shows that Atlantic salmon are more efficient, steady-endurance swimmers. When swimming in wave-like unsteady flow conditions, they use twice as much energy, contrary to some pectoral fin swimmers that are highly skilled in station holding and manoeuvring [3,4].

Finally, based on the ODBA/MO_2_ regression equations, the standard metabolic rate of Atlantic salmon in steady and unsteady flow at zero acceleration was estimated to be 168 and 180 mg O_2_ kg^−1^ h^−1^, respectively. This is similar to the results obtained by Hvas et al. [56] for fish swimming at 13 °C under steady flow conditions. The positive linear correlation between ODBA and MO_2_ observed for Atlantic salmon swimming in steady flow was also similar to what was observed for Sockeye salmon (*Oncorhynchus nerka*) (root mean square acceleration and MO_2_; R^2^ = 0.87) swimming under the same flow condition and temperature [31]. Gleiss and colleagues [28] found a linear correlation (R^2^ > 0.71) between MO_2_ and partial dynamic body acceleration (PDBA_yz_: only heave and sway axes used in calculation) when scalloped hammerhead (*Sphyrna lewini*) swam in a respirometer.

Together, these findings imply that the sensor tags used with ODBA settings may prove useful in offshore salmon farms to infer the real-time energy expenditure of cultured fish, providing insight into their capacity to cope with various biological and climate stressors.

## 5. Conclusions

The acoustic transmitter tags did not alter any of the quantified parameters in our study of Atlantic salmon and can be used to monitor swimming performance under wave-like, unsteady flow conditions. This study demonstrated that swimming in unidirectional, unsteady flow is energetically more costly for post-smolt Atlantic salmon than swimming in steady flow. ODBA predicts MO_2_ well in both steady and unsteady flows. Therefore, acoustic transmitter tags with ODBA settings have the potential to be used to estimate the real-time activity-related energy expenditure of fish in both aquaculture and nature.

## Figures and Tables

**Table 1:** Biometrical parameters of experimental fish (*Salmo salar*) at the start of the swim test. Standard length (SL), body weight (BW), and condition factor (K) are shown for fish that were implanted with an acceleration tag (Tag) or not (Non-tag), swimming under steady or unsteady flow conditions. Values are reported as the mean ± SE. Significant differences (ANOVA, *p* < 0.05) between groups are indicated by different letters.

	Non-Tag Steady (Group 1)	Non-Tag Unsteady (Group 2)	Tag Steady (Group 3)	Tag Unsteady (Group 4)
N	10	10	10	10
SL	28.3 ± 0.4 ^a,b^	28.3 ± 0.2 ^a^	26.9 ± 0.3 ^b^	26.8 ± 0.5 ^b^
BW	315 ± 15 ^a^	318 ± 6 ^a^	275 ± 8 ^b^	265 ± 8 ^b^
K	1.49 ± 0.05 ^a^	1.40 ± 0.03 ^a^	1.40 ± 0.03 ^a^	1.38 ± 0.04 ^a^

## Data Availability

Raw data supporting the conclusions of this article will be made available by the authors, without undue reservation, to any qualified researcher.

## Supplementary Materials

The following supporting information can be downloaded at: https://www.mdpi.com/article/10.3390/biology13060393/s1, Figure S1: Flow speed calibrations; Figure S2: Oxygen consumption (MO_2_) of non-tagged fish (*Salmo salar*) at increasing swimming speed in both steady and unsteady flow; Figure S3: Tail beat frequency, tailbeat amplitude, head width frequency, and head width amplitude vs. swimming speed for non-tagged post-smolt Atlantic salmon (*Salmo salar*) swimming in steady and unsteady flow; Table S1: Linear mixed model (LMM) results for the effect of swimming speed, flow condition, and tag on oxygen consumption (MO_2_), cost of transport (COT), tail beat frequency (TBF), tail beat amplitude (TBA), head width frequency (HWF), head width amplitude (HWA), and overall dynamic body acceleration (ODBA); Table S2: Regression equation, goodness of fit (R^2^), and ranges of locomotory parameters measured with a high-speed camera during a critical swimming speed protocol.
