# Supplementary material for: Swimming at Increasing Speeds in Steady and Unsteady Flows of Atlantic Salmon Salmo salar: Oxygen Consumption, Locomotory Behaviour and Overall Dynamic Body Acceleration
Source: Biology (Basel). 2024 May 29;13(6):393. doi: 10.3390/biology13060393 (PMC11200746; doi:10.3390/biology13060393)
Supplement: Supplementary file 1 [file biology-13-00393-s001.zip › biology-3016825-supplementary.pdf]

Table S1. Linear mixed model (LMM) results for effect of swimming speed, flow condition and tag on oxygen consumption (MO2), cost of transport (COT), tail beat frequency (TBF), tail beat amplitude (TBA), head width frequency (HWF), head width amplitude (HWA) and overall dynamic body acceleration (ODBA). Individual variability was accounted for as random effect in each model. Intercept, Beta estimate and R^2 values are provided for each model. Standardised parameters were obtained by fitting the model on a standardised version of the dataset. 95% confidence intervals (CIs) and p-values were computed using a Wald t-distribution approximation.

Model	Estimate (β)	Value [95% CI]	*p*	t	R^2 marginal	R^2 conditional
MO_2 ~ swim speed + tag	Intercept	175 [31, 318]	<0.001	t(140) = 12.9	0.46	0.61
	0.4 $m.s^{-1}$	-10 [-42, 23]	0.56	-0.6		
	0.6 $m.s^{-1}$	103 [71, 136]	<0.001	6.3		
	0.8 $m.s^{-1}$	158 [121, 194]	<0.001	8.6		
	unsteady	89 [51, 126]	<0.001	4.7		
	tagged	-26 [-63, 12]	0.18	-1.4		
COT ~ swim speed + flow type + tag	Intercept	363 [333, 394]	<0.001	t(140) = 23.6	0.66	0.74
	0.4 $m.s^{-1}$	-199 [-227, -172]	<0.001	-14.3		
	0.6 $m.s^{-1}$	-209 [-236, -181]	<0.001	-14.9		
	0.8 $m.s^{-1}$	-233 [-263, -202]	<0.001	-14.9		
	unsteady	59 [29, 89]	<0.001	3.9		
	tagged	-16 [-46, 14]	0.28	-1.1		
TBF ~ swim speed + flow type + tag	Intercept	4.1 [3.2, 5.0]	<0.001	t(119) = 10.0	0.30	0.49
	0.4 $m.s^{-1}$	-0.2 [-0.9, 0.5]	0.57	-0.6		
	0.6 $m.s^{-1}$	0.6 [-0.1, 1.4]	0.08	1.8		
	0.8 $m.s^{-1}$	2.9 [2.1, 3.8]	<0.001	7.0		
	unsteady	-0.1 [-0.9, 0.7]	0.80	-0.3		
	tagged	-0.05 [-0.8, 0.7]	0.90	-0.1		
TBA ~ swim speed + flow type + tag	Intercept	0.5 [0.3, 0.7]	<0.001	t(114) = 5.3	0.55	0.65
	0.4 $m.s^{-1}$	0.6 [0.4, 0.7]	<0.001	6.0		
	0.6 $m.s^{-1}$	1.0 [0.8, 1.2]	<0.001	10.5		
	0.8 $m.s^{-1}$	1.1 [0.9, 1.3]	<0.001	10.6		
	unsteady	0.3 [0.1, 0.5]	<0.001	3.5		
	tagged	0.2 [-0.03, 0.4]	0.10	1.7		
HWF ~ swim speed + flow type + tag	Intercept	3.1 [2.3, 3.9]	<0.001	t(130) = 7.4	0.45	0.61
	0.4 $m.s^{-1}$	1.5 [0.8, 2.2]	<0.001	4.2		
	0.6 $m.s^{-1}$	2.6 [1.9, 3.3]	<0.001	7.2		
	0.8 $m.s^{-1}$	4.6 [3.9, 5.4]	<0.001	11.9		
	unsteady	-0.9 [-1.8, -0.01]	0.07	2.3		
	tagged	0.3 [-0.4, 1.4]	0.36	-0.3		
HWA ~ swim speed + flow type + tag	Intercept	5.9 [5.2, 6.6]	<0.001	t(122) = 15.9	0.17	0.29
	0.4 $m.s^{-1}$	0.04 [-0.7, 0.8]	0.91	0.1		
	0.6 $m.s^{-1}$	1.6 [0.8, 2.3]	<0.001	4.2		
	0.8 $m.s^{-1}$	1.6 [0.7, 2.4]	<0.001	3.6		
	unsteady	-0.07 [-0.8, 0.6]	0.84	-0.2		
	tagged	-0.2 [-0.9, 0.5]	0.64	-0.5		

log_ODBA ~ swim speed × flow type	Intercept	-0.56 [-0.64, -0.47]	<0.001	t(2523) = -13.1	0.27	0.37
	0.4 $m.s^{-1}$	0.07 [0.02, 0.11]	0.003	2.9		
	0.6 $m.s^{-1}$	0.33 [0.28, 0.37]	<0.001	5.9		
	0.8 $m.s^{-1}$	0.41 [0.35, 0.47]	<0.001	5.9		
	unsteady	0.04[-0.08, 0.15]	0.61	-1.1		
	0.4 $m.s^{-1}$ × unsteady	0.14 [0.07, 0.20]	<0.001	4.1		
	0.6 $m.s^{-1}$ × unsteady	0.17 [0.1, 0.23]	<0.001	6.3		
	0.8 $m.s^{-1}$ × unsteady	0.003[-0.08, 0.07]	0.94	2.8		
MO_2 ~ log_ODBA + swim speed * flow type	intercept	265 [225, 306]	<0.001	t(2522) = 12.8	0.49	0.77
	log_ODBA	19 [12, 27]	<0.001	5.2		
	0.4 $m.s^{-1}$	-31 [-39, 29]	<0.001	-7.4		
	0.6 $m.s^{-1}$	10 [2, 19]	<0.001	2.3		
	0.8 $m.s^{-1}$	82 [70, 93]	<0.001	14.2		
	unsteady	29 [-28, 86]	0.32	1.0		
	0.4 $m.s^{-1}$ × unsteady	36 [24, 47]	<0.001	6.1		
	0.6 $m.s^{-1}$ × unsteady	108 [97, 120]	<0.001	18.2		
	0.8 $m.s^{-1}$ × unsteady	102 [88, 115]	<0.001	14.3		

Table S2. Regression equation, goodness of fit (R^2) and ranges of locomotory parameters measured with a high-speed camera during a critical swimming speed protocol. Range of values for each parameter per experimental group are reported from 0.2 to 0.8 $m.s^{-1}$ in their respective units.

Locomotory parameters	Experimental groups	Range of value parameters	Goodness of fit (R^2)
**TBF**	Tag unsteady	4.03 ± 0.43 to 6.12 ± 0.8 $cycle.s^{-1}$	0.92
	Tag steady	4.36 ± 0.61 to 7.02 ± 1.52 $cycle.s^{-1}$	0.86
	Non-tagged unsteady	3.59 ± 0.26 to 6.65 ± 0.30 $cycle.s^{-1}$	0.98
	Non-tagged steady	3.72 ± 0.19 to 7.32 ± 0.92 $cycle.s^{-1}$	0.91
**TBA**	Tag unsteady	1.03 ± 0.16 to 2.20 ± 0.18 cm	0.99
	Tag steady	0.65 ± 0.15 to 1.90 ± 0.15 cm	0.98
	Non-tagged unsteady	0.86 ± 0.09 a.u. to 2.06 ± 0.42 cm	0.96
	Non-tagged steady	0.50 ± 0.08 a.u. to 1.73 ± 0.13 cm	0.95
**HWF**	Tag unsteady	2.49 ± 0.22 to 6.53 ± 0.76 $cycle.s^{-1}$	0.98
	Tag steady	2.69 ± 0.33 to 7.56 ± 1.06 $cycle.s^{-1}$	0.97
	Non-tagged unsteady	2.59 ± 0.18 to 5.65 ± 1.06 $cycle.s^{-1}$	0.94
	Non-tagged steady	2.44 ± 0.28 to 8.65 ± 0.94 $cycle.s^{-1}$	0.94
**HWA**	Tag unsteady	5.40 ± 0.63 to 7.35 ± 0.43 cm	0.83
	Tag steady	5.80 ± 0.3 to 7.92 ± 1.72 cm	0.87
	Non-tagged unsteady	6.01 ± 0.51 to 8.41 ± 0.30 cm	0.98
	Non-tagged steady	5.51 ± 0.46 to 7.67 ± 0.90 cm	0.86

(A)

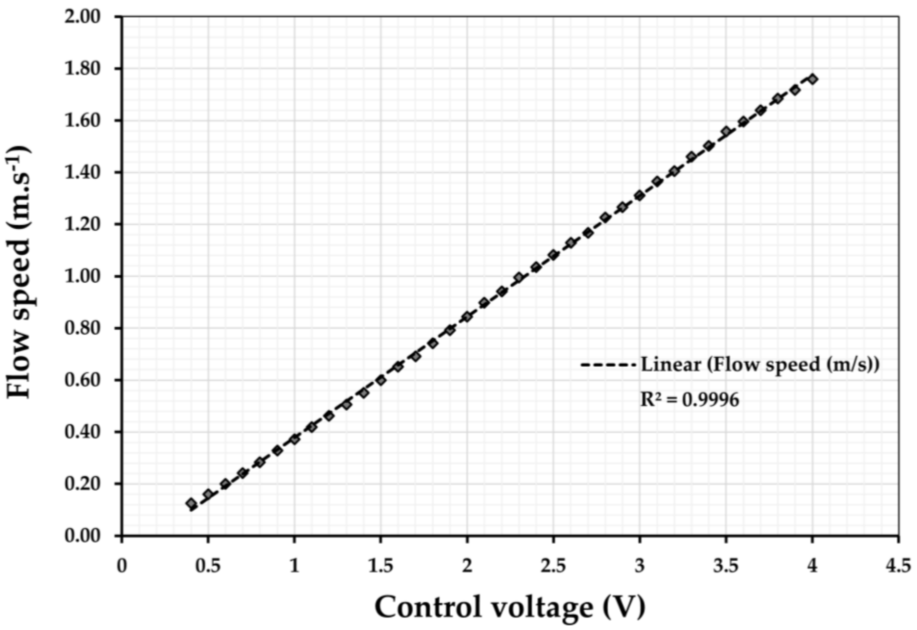

(B)

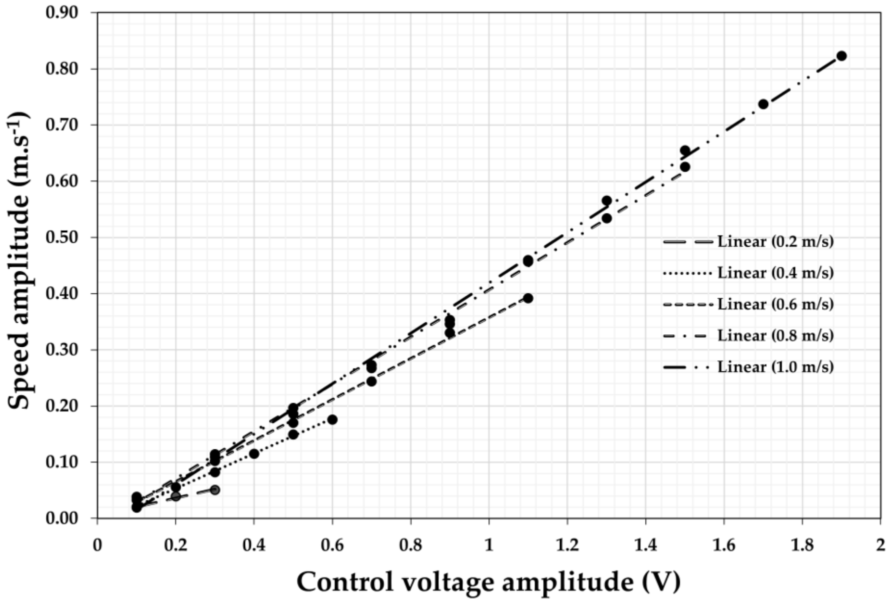

Figure S1. Flow speed calibrations. (A) Steady flows. The dashed line represents a linear regression of flow speed vs. control voltage; (B) Sinusoidal modulations; speed modulation amplitudes were measured for voltage amplitude modulations in steps of 0.1 V, at different mean flow speeds (see legend) and a modulation period of 12 s. Linear regression lines were used to determine the voltage modulation required for a speed modulation of 0.1 m/s at each mean flow speed.

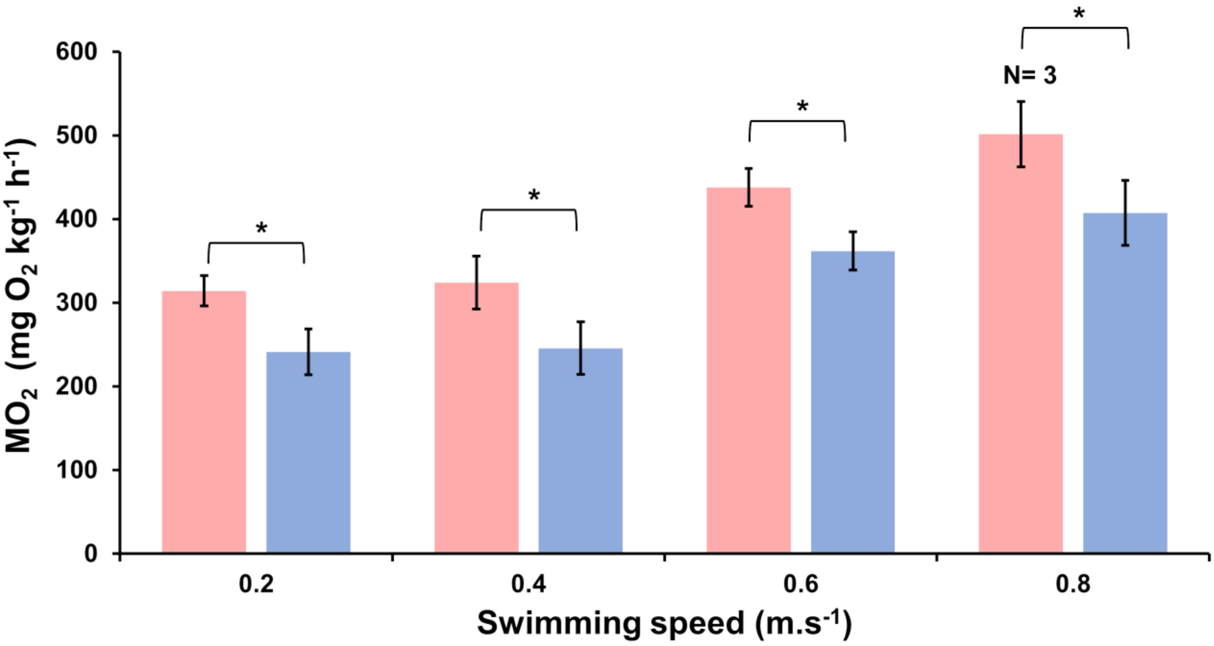

Figure S2: Oxygen consumption (MO_2) of non-tagged fish (*Salmo salar*) at increasing swimming speed in both steady and unsteady flow. The red colour represents swimming in unsteady flow, the blue colour swimming in unsteady flow. Each bar represents N= 10 fish unless indicated otherwise because fish had fatigued. Asterisk indicates significant difference (LMM $p < 0.05$) between steady and unsteady flow. MO_2 values are shown as means ±SE.

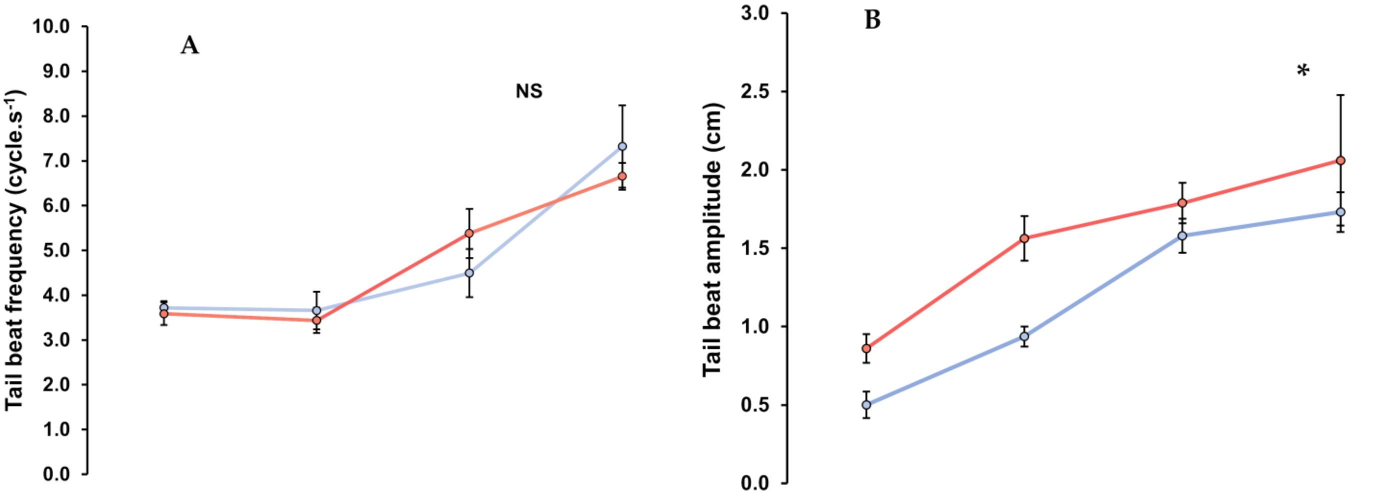

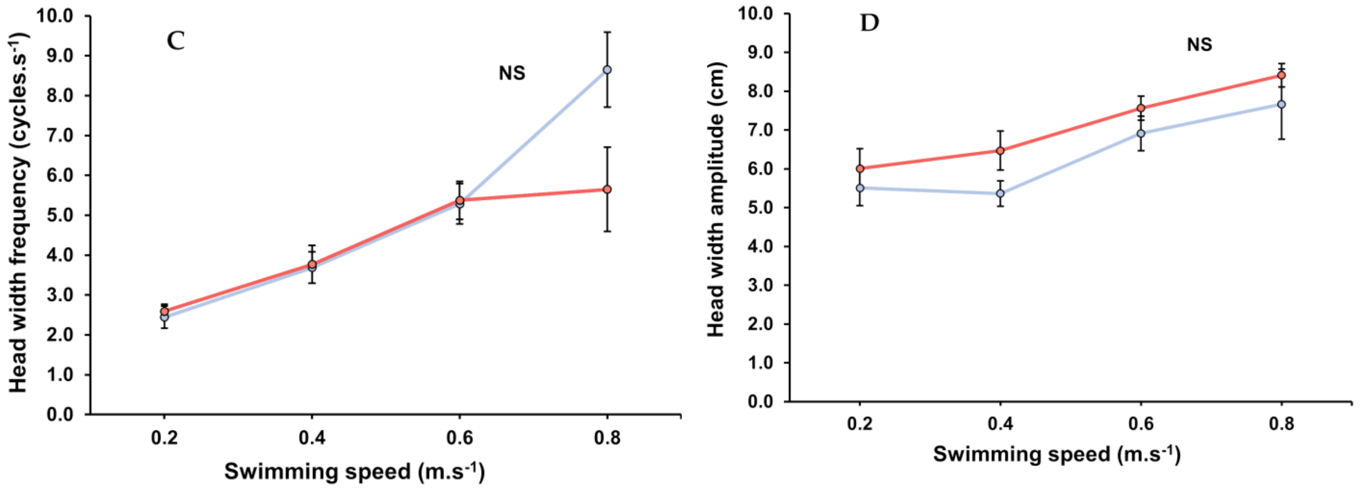

Figure S3. Tail beat frequency, tailbeat amplitude, head width frequency, and head width amplitude vs. swimming speed for non-tagged post-smolt Atlantic salmon (*Salmo salar*) swimming in unsteady and steady flow with A) Tail beat frequency vs. swimming speed; B) Tail beat amplitude vs. swimming speed, C) Head width frequency vs. swimming speed, and D) Head width amplitude vs. swimming speed. The red colour represents fish swimming in unsteady flow, while the blue colour represents fish swimming in steady flow. Asterisk indicates significant difference (LMM $p < 0.05$) between steady and unsteady flow, while NS indicate no significant difference.
